# Climatic and Catchment-Scale Predictors of Chinese Stream Insect Richness Differ between Taxonomic Groups

**DOI:** 10.1371/journal.pone.0123250

**Published:** 2015-04-24

**Authors:** Jonathan D. Tonkin, Deep Narayan Shah, Mathias Kuemmerlen, Fengqing Li, Qinghua Cai, Peter Haase, Sonja C. Jähnig

**Affiliations:** 1 Department of River Ecology and Conservation, Senckenberg Research Institute and Natural History Museum Frankfurt, Gelnhausen, Germany; 2 Biodiversity and Climate Research Centre (BiK-F), Frankfurt am Main, Germany; 3 State Key Laboratory of Freshwater Ecology and Biotechnology, Institute of Hydrobiology, Chinese Academy of Sciences, Wuhan, P.R. China; 4 Leibniz-Institute of Freshwater Ecology and Inland Fisheries (IGB), Department of Ecosystem Research, Berlin, Germany; Aberystwyth University, UNITED KINGDOM

## Abstract

Little work has been done on large-scale patterns of stream insect richness in China. We explored the influence of climatic and catchment-scale factors on stream insect (Ephemeroptera, Plecoptera, Trichoptera; EPT) richness across mid-latitude China. We assessed the predictive ability of climatic, catchment land cover and physical structure variables on genus richness of EPT, both individually and combined, in 80 mid-latitude Chinese streams, spanning a 3899-m altitudinal gradient. We performed analyses using boosted regression trees and explored the nature of their influence on richness patterns. The relative importance of climate, land cover, and physical factors on stream insect richness varied considerably between the three orders, and while important for Ephemeroptera and Plecoptera, latitude did not improve model fit for any of the groups. EPT richness was linked with areas comprising high forest cover, elevation and slope, large catchments and low temperatures. Ephemeroptera favoured areas with high forest cover, medium-to-large catchment sizes, high temperature seasonality, and low potential evapotranspiration. Plecoptera richness was linked with low temperature seasonality and annual mean, and high slope, elevation and warm-season rainfall. Finally, Trichoptera favoured high elevation areas, with high forest cover, and low mean annual temperature, seasonality and aridity. Our findings highlight the variable role that catchment land cover, physical properties and climatic influences have on stream insect richness. This is one of the first studies of its kind in Chinese streams, thus we set the scene for more in-depth assessments of stream insect richness across broader spatial scales in China, but stress the importance of improving data availability and consistency through time.

## Introduction

Describing and explaining large-scale patterns of species richness has long been a focus of ecological and biogeographical research [1–3]. Many clear biodiversity patterns exist in nature, such as the latitudinal diversity gradient, which has been observed in various taxonomic groups resulting from a suite of potential influences [4]. Among other factors, much of the geographical variation in species distributions can be related to either climatic (e.g. mean annual temperature, precipitation, potential evapotranspiration) or historical (e.g. ice age glaciations, dispersal) influences [1,4].

Streams provide unique testing grounds for biodiversity assessments at large spatial scales due to their similarity in local habitat conditions globally [5], in addition to the fact that factors such as climate may operate on running water systems in a different manner to terrestrial ecosystems [6]. Factors that influence and control the biodiversity of stream insects vary greatly and differ with scale [5,7], such as present and past land use [8,9], productivity [10], climate change [11], the flow regime [12], and temperature [13]. However, what remains clear is that our understanding of large-scale biodiversity patterns and their drivers is very much incomplete [7].

While stream invertebrate communities have predominantly been assessed in relation to local-scale factors (those that exhibit variation at small spatial scales), with less focus on broader patterns [14], attention has increasingly shifted in recent times towards larger- or multi-scale factors, including regional species pools [15–18]. However, the results have been equivocal for streams with regard to the importance of local versus regional drivers (e.g. [6]), but it is likely that stream communities are determined by a suite of factors operating at multiple spatial scales and depending on the scale of observation [16,18–20].

China’s large geographical area and diverse topography, with about two thirds of its landmass covered by mountains, provides broad environmental gradients and unique evolutionary history, which translates into high levels of biodiversity [21–23]. However, the rapid economic development and increasing intensity of anthropogenic disturbances to landscapes and river basins have significantly altered land use [24,25] and hydrological processes (e.g. flow regime); these in turn have led to a severe loss of freshwater species [21].

Despite the rich biodiversity of Chinese freshwater environments and in line with stream biodiversity in general, our knowledge is limited with regard to the large-scale influences on stream insect richness. While there are many smaller-scale studies (e.g. [26,27]), large-scale studies on freshwater organisms, particularly for stream insects, are essentially lacking in China (but see [28]). Therefore, we set out to examine drivers of stream insect richness in three regions across mid-latitude China. We focused on three orders of stream insects, Ephemeroptera (E; mayflies), Plecoptera (P; stoneflies) and Trichoptera (T; caddisflies), as well as their combined richness (EPT). EPT are commonly used for answering fundamental ecological questions [5], and for bioassessment purposes due to their sensitivity and ability to respond consistently to environmental conditions [29]. With these knowledge gaps of stream insect biodiversity in mind, particularly for China, we took an exploratory approach to identifying the key climatic and catchment-scale influences on stream insect (EPT) richness. In particular, we asked the following questions: 1) Which climatic and catchment-scale variables, including land cover and physical structure, best predict EPT genus richness patterns in mid-latitude China? In what manner do these variables influence richness? 3) Are the three different insect orders predicted by a different set of variables?

## Materials and Methods

### Site selection

**Fig 1:**
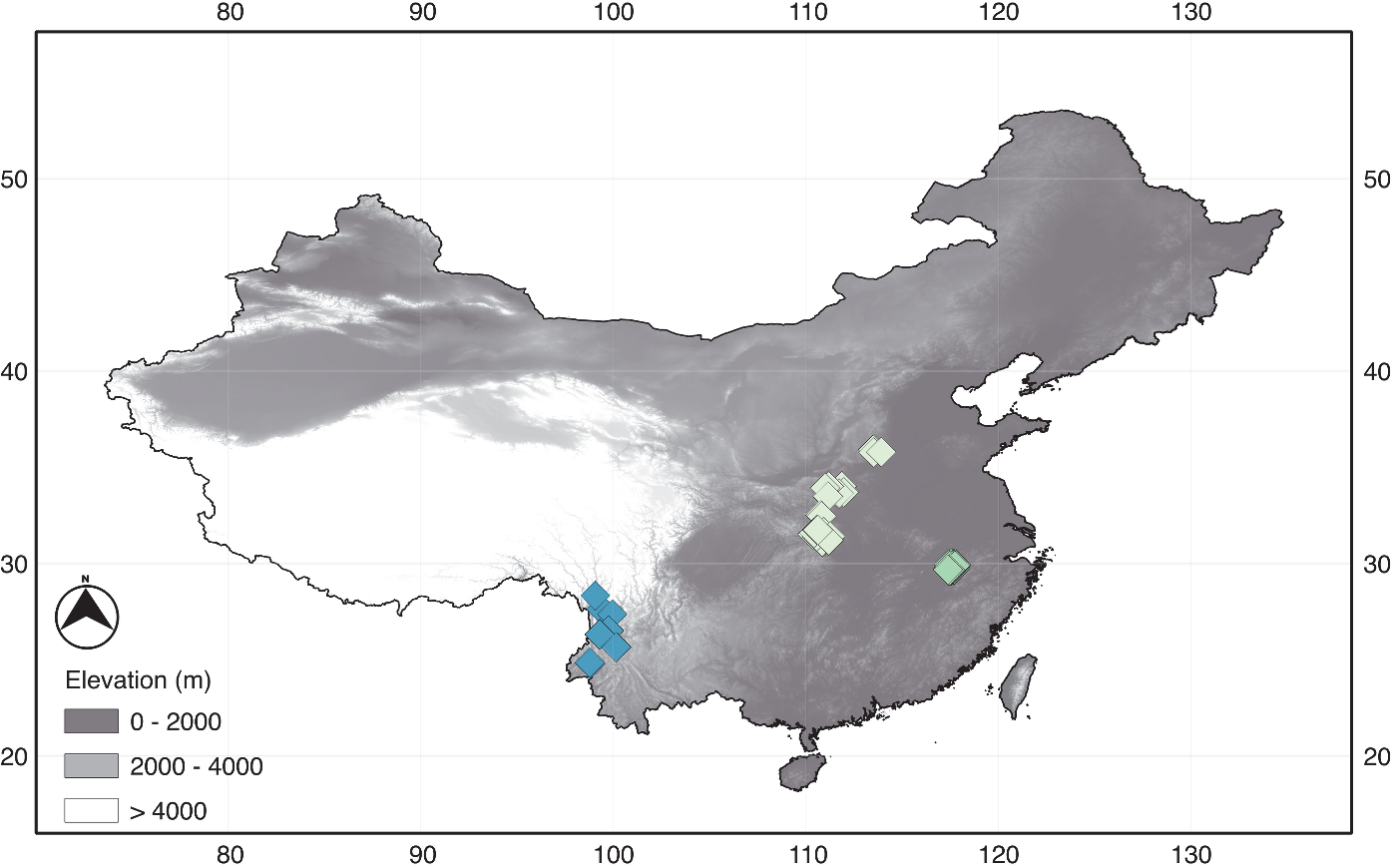
Map of study sites. Map showing the location of the 80 sampling sites across mid-latitude China used in the study. Values on the horizontal and vertical axes are degrees east and north respectively.

Eighty sites were included in the study from three locations (West: 10 sites; Central: 29 sites; East: 41 sites) in the mid-latitudes of China between 25° and 36° N latitude, 98° and 118° longitude (Fig 1), and with an altitudinal gradient of 3899 m (min: 65; max: 3964 m asl; S1 Fig), spanning from the western Chinese Province of Yunnan to the Anhui province in the east. All the sites were selected to be relatively unimpaired sites, in near-natural conditions, based on present-day conditions. Most were located in landscapes dominated by forest, with limited agricultural and urban land cover.

Where permission to sample was required, it was granted by Cangshan, Baimaxueshan and Gaoligongshan Nature Reserve Administrations (some western sites), and Hubei Shennongjia National Nature Reserve Bureau (some central sites). All other sites were outside of nature reserves and thus no specific permission was required to sample the streams, given no endangered or protected species were collected or involved in sampling.

### Biological data

Sampling was performed between November 2006 and October 2011. Sampling approaches and times differed between regions (West: October 2011; Central: November 2006 and April 2008; East: November 2010). Some of these data have been used in previous publications [28,30–32]. However, sampling was performed to reflect habitat composition adequately. Macroinvertebrate samples were collected from a 50 to 100-m reach of the selected stream. Sampling at most sites followed a standardized multihabitat sampling approach [33], where between 10 and 20 subsamples representing the current habitat composition (total sampling area 1.25 m^2^) were taken with a 500-μm shovel sampler. Eleven of the 29 central sites were collected using three 0.1-m^2^ Surber sampler, but EPT richness did not differ between the two approaches within this region (Mann-Whitney U = 96.5, *P* = 0.928), so we included these 11 sites in the analysis. A single composite sample was made and samples were preserved with 75–95% alcohol (or 10% formalin in the 11 aforementioned central sites) in the field and sorted and identified in the laboratory to genus level where possible (with the exception of very few difficult-to-identify taxa) using available keys [34–38].

### Environmental variables

To predict richness of EPT we used three categories of environmental variables: bioclimatic (based on immediate location of sampling site), catchment land cover (based on percentage of upstream land cover categories), and physical properties (based on elevation and catchment slope and size). To do this, we created watersheds for each site with the ‘watershed’ tool in ArcMap 10.0 (ESRI Inc., Redlands, CA, U.S.A.), using the ‘flow direction’ layer of 30 arc-seconds (~1 km) resolution from the HydroSHEDS database [39] (http://hydrosheds.cr.usgs.gov). The catchment size in km^2^ was calculated by summing up the number of grids (1 × 1 km). Catchment slope was also calculated in ArcMap 10.0.

Present climatic predictors with a spatial resolution of 30 arc-seconds (approximately 1 km; Worldclim; http://www.worldclim.org; accessed 25 March 2014) [40] were extracted for each sampling site using the ‘sample’ tool of ArcMap 10.0 (Table 1). Global Aridity Index (AI) and Global Potential Evapo-Transpiration (PET) data (CGIAR Consortium for Spatial Information; http://www.csi.cgiar.org) [41] were extracted for each sample point in the same manner (Table 1).

**Table 1:** Environmental variables included in analyses.

**Category**	**Code**	**Value**
**Climate**	**BIO1**	Annual Mean Temperature
	BIO2	Mean Diurnal Range (Mean of monthly (max temp—min temp))
	BIO3	Isothermality (BIO2/BIO7) (x 100)
	**BIO4**	Temperature Seasonality (SD x 100)
	BIO5	Max Temperature of Warmest Month
	BIO6	Min Temperature of Coldest Month
	BIO7	Temperature Annual Range (BIO5-BIO6)
	**BIO8**	Mean Temperature of Wettest Quarter
	BIO9	Mean Temperature of Driest Quarter
	BIO10	Mean Temperature of Warmest Quarter
	BIO11	Mean Temperature of Coldest Quarter
	BIO12	Annual Precipitation
	BIO13	Precipitation of Wettest Month
	BIO14	Precipitation of Driest Month
	**BIO15**	Precipitation Seasonality (Coefficient of Variation)
	BIO16	Precipitation of Wettest Quarter
	BIO17	Precipitation of Driest Quarter
	**BIO18**	Precipitation of Warmest Quarter
	BIO19	Precipitation of Coldest Quarter
	**PET**	Potential Evapo-transpiration
	**AI**	Aridity Index
**Land cover**	**Trees (BL)**	% catchment broadleaf trees
	**Trees (NL)**	% catchment needleleaf trees
	**Shrub**	% catchment shrub vegetation
	**Herbaceous**	% catchment herbaceous vegetation
	**Cultivated**	% catchment cultivated land cover
	**Water**	% catchment water
**Physical**	**Elevation**	Elevation in m asl
	**Catchment size**	Catchment size in km^2^
	**Slope**	Catchment slope in degrees
	**Region**	Region (west, central, east)

Environmental variables used to predict stream insect richness in 80 streams across mid-latitude China. Autocorrelated bioclimatic variables (*r* > 0.75) were excluded from the analyses Variables selected to predict richness are given in bold. BIO values are BIOCLIM parameters. Temperature values are measured in °C and multiplied by 10, precipitation values are in mm. All other units or transformations are given in the table.

Land cover data (Global Land Cover Database; http://bioval.jrc.ec.europa.eu/products/glc2000/products.php, accessed 16 August 2013) was extracted from the entire upstream catchment of each site (Table 1). The percentage of present land cover was calculated for the following land cover types: broadleaf trees, needleleaf trees, shrub, herbaceous, cultivated, and water (Table 1). The cumulative land cover of the upper subcatchment upstream from individual sites has been found to be a better predictor of species occurrence than the land use at the sampling site itself [9,42].

### Statistical analyses

All statistical analyses were carried out in R 3.0.2 [43].

To characterise and compare insect richness patterns and environmental variables between each of the three regions, we tested for differences using a combination of linear and generalized linear modeling (GLM). First, we tested for differences in environmental variables between the three regions with one-way ANOVA using the ‘aov’ function (S1 Fig). Second, we tested for normality of richness of each of the groups and EPT combined using the Shapiro-Wilk test of normality with the ‘shapiro.test’ function, followed by visual inspection of qq-plots. As richness was not normally distributed, we used GLMs with Poisson error distribution and log link functions to test for differences between the three regions. We followed these up with posthoc pairwise Tukey's tests to test for differences between individual groups, using the 'glht' function in the 'multcomp' package [44].

#### Boosted regression trees

To explore the influence of the environmental variables on stream insect richness for both combined and individual EPT groups, we used boosted regression trees (BRT) [45–47] in the package ‘dismo’ [48]. BRT is a powerful and advanced form of regression, which combines traditional statistical techniques with machine learning. The boosting approach of BRT enhances predictive ability by combining a large number of relatively simple regression trees [47,49], as opposed to producing a single optimal (complex) model of traditional least squares regression. BRT excels in its ability to model complex non-linear relationships, and is thus ideal for identifying key predictor variables.

We aimed to extract the most important drivers of richness, thus we used the gbm.step procedure, which takes a stepwise approach to model selection, and we used the poisson family loss function. Each successive tree is fitted to the residuals of those already selected, and shrinking is used to reduce the contribution of each tree and averaged across the final selected set of trees. For selection of the optimal number of trees, we used 10-fold cross-validation. Cross validation here allows testing the developing model with held-out data, ensuring a final model that is able to predict non-training data. We set the bag fraction of models to 0.5, which introduces randomness into the model, by randomly selecting a fraction of the training set to propose for the next tree. We used a learning rate of 0.0005 to ensure at least 1000 trees were reached, which is a relatively slow learning rate ensuring reliable model estimates. We set tree complexity to 5, which controls the number of splits in individual trees.

Given the lack of latitudinal range in this study and the fact the three regions were well separated, as well as the strong west to east gradient of many of the environmental variables such as elevation, slope and rainfall, we performed two groups of analyses separately: one including and one not including latitude as a predictor variable (eight models total). This allowed us to tease apart the importance of latitude and the underlying environmental gradients driving patterns in richness. Furthermore, because of the clear grouping of sites into three distinct regions, we also included a variable ‘region’ into the models, to account for any potentially unmeasured or historic influences unique to the three regions.

For each of these two approaches, we used the same settings for each of the four models (EPT, E, P, T) and included all environmental variables from each of the three categories (climate, land cover and physical) in each model (Table 1). While gradient boosting methods are robust towards collinear predictor variables, to aid interpretability, we removed highly correlated variables (*r* > 0.75). This resulted in 7 of the original 21 bioclimatic variables being used (Table 1).

The relative influence of individual predictors can be assessed in BRT, based on how often the variable is selected, and how this selection improves the model. These relative influences are then scaled so the sum of influences equal 100%. We also investigated the shape of the fitted functions of the top five variables influencing each of the four models using partial dependence plots. These plots indicate the model outcome in relation to the given independent variable, after considering the average effect of other independent variables in the model. We estimated overall model performances with cross-validated percentage of deviance explained, and the cross-validated correlation coefficient between observed and model-fitted values. While we present both the training data and cross-validated results, the training data results should not be evaluated as they have likely overfit the data.

## Results

**Fig 2:**
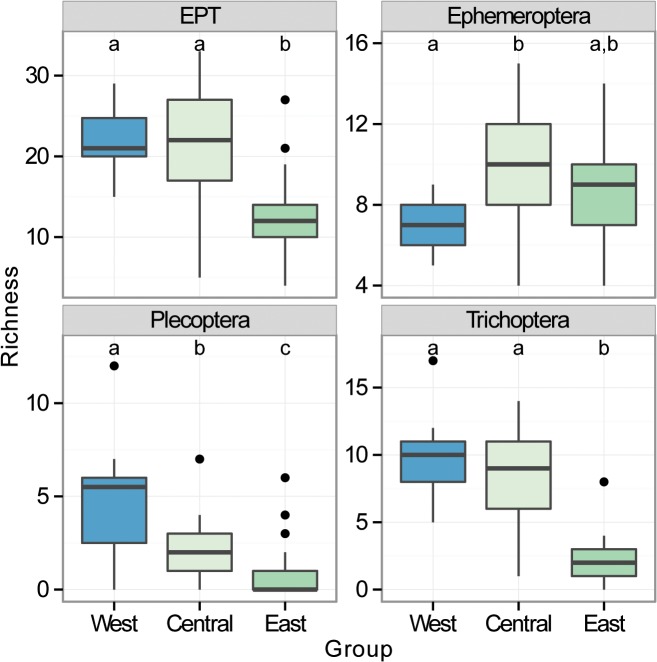
Summary of regional richness. Summary of Ephemeroptera (E), Plecoptera (P), Trichoptera (T) and combined EPT richness (mostly genus level) between the three regions taken from 80 streams across mid-latitude China. Results of pairwise Tukey's posthoc tests on generalized linear models are shown as letters at the top of each plot. Different letters represent significant differences between regions. Boxes represent the interquartile range (IQR), and whiskers are the furthest point within 1.5 x IQR above or below the IQR. Values beyond this range are plotted as individual points. The central line represents the median.

EPT richness ranged between 4 and 33 taxa, with a mean of 18.1 ± 0.8 (S.E.), and was significantly lower in eastern sites (Fig 2; *Χ* ^2^ = 136.25, D.F. = 2,77, *P* < 0.0001). Ephemeroptera richness averaged 9.4 ± 0.3, varying between 4 and 15 taxa and was lower in western sites compared to the central but not eastern sites (Fig 2; *Χ* ^2^ = 59.57, D.F. = 2,77, *P* = 0.02). Plecoptera richness, on the other hand, was much lower across sites (0–12 taxa; mean = 2.6 ± 0.3), and Trichoptera were in between but more variable (0–17 taxa; mean = 6.1 ± 0.5; Fig 2). Plecoptera richness declined from west to east (*Χ* ^2^ = 140.49, D.F. = 2,77, *P* < 0.0001) and Trichoptera (*Χ* ^2^ = 97.99, D.F. = 2,77, *P* < 0.0001) richness was lower in the eastern compared to the western and central sites (Fig 2).

### Boosted regression trees

#### Important environmental variables

**Fig 3:**
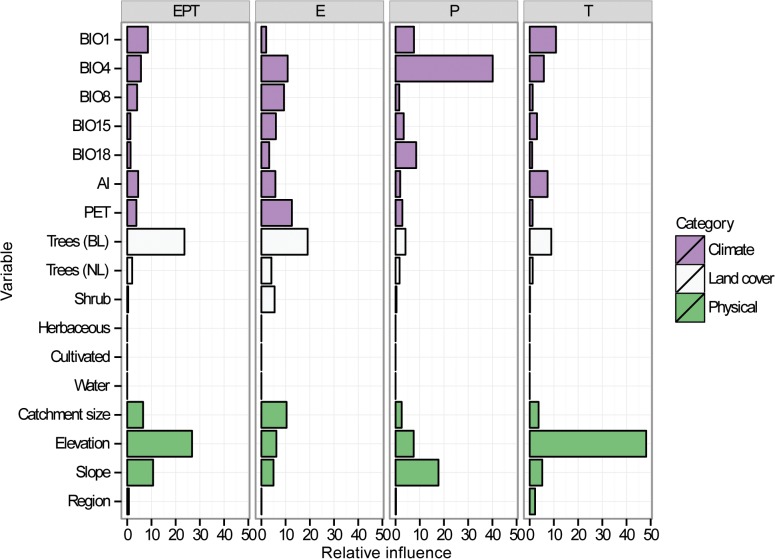
Relative influence of variables on richness patterns. Relative influence of each of the variables (climate, land cover and physical) on each of the four boosted regression tree models not including latitude predicting Ephemeroptera (E), Plecoptera (P), Trichoptera (T) and combined EPT richness taken from 80 streams across mid-latitude China. Explanations and units of variables are given in Table 1.

**Fig 4:**
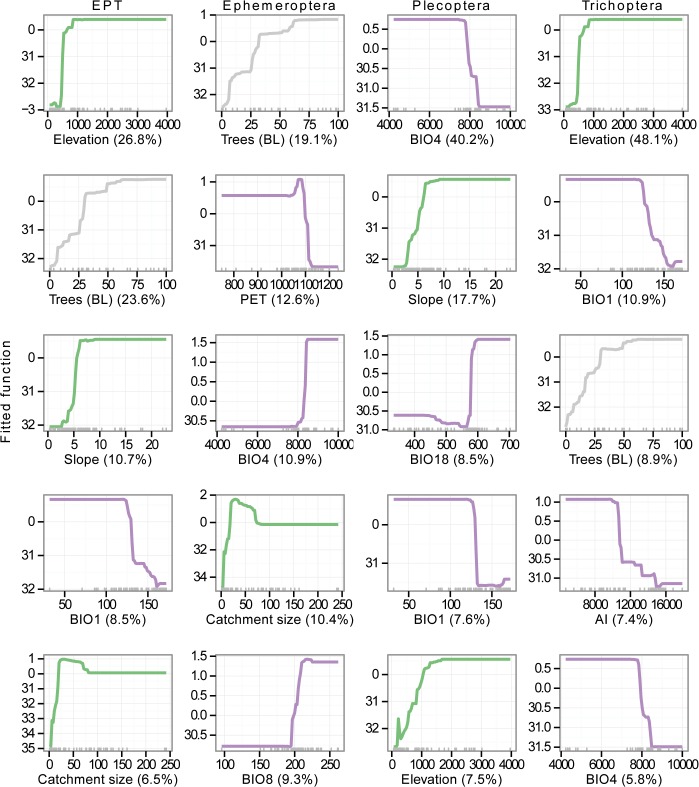
Partial dependence plots of most important variables. Partial dependence plots showing fitted functions of each of the top five influential variables contributing to each of the four boosted regression tree models not including latitude predicting Ephemeroptera (E), Plecoptera (P), Trichoptera (T) and combined EPT richness from environmental variables in streams across mid-latitude China. Y-axis values represent the model outcome in relation to the given independent variable, after considering the average effect of other independent variables in the model. For consistency between all independent variables, influence values have been scaled to have a mean = 0 and standard deviation = 1. Percentages given in parentheses next to variable names are the relative influence on the model. Rugs along the x-axis represent the data points. Climate variables: purple; land cover variables: grey; physical variables: green. Explanations and units of variables are given in Table 1. Note temperature values are in °C x 10.

Climatic, physical and land cover variables all influenced richness of the four groupings (Table 2 and Figs 3 and 4). The only land cover variable to have a strong input in the models was percentage of catchment in broadleaf tree cover (Figs 3 and 4). Slope, elevation and catchment size all contributed substantially to individual models (Figs 3 and 4). Despite significant variation in climatic variables between the three regions (S1 Fig), no climatic variables had consistently strong influences for the three taxonomic groups (Figs 3 and 4). However, temperature variables were consistently more important than precipitation (Figs 3 and 4). Inclusion of latitude in the models did not improve model fit for any of the models (Table 2). Furthermore, the factor ‘region’ had little influence on any of the models.

**Table 2:** Results of boosted regression trees. Results of the eight boosted regression tree models predicting genus richness of Ephemeroptera (E), Plecoptera (P), Trichoptera (T) and combined EPT from climatic, catchment land cover and physical variables in 80 streams across mid-latitude China. The first four models do not include latitude as a predictor. N trees = number of trees; dev = deviance.

Model Summary Information			Training model				Cross validated		
Latitude included	Group	N trees	% dev explained	Correlation	Mean null dev	Mean residual dev	% dev explained	Correlation	Estimated dev
No	EPT	5700	67.9	0.840	2.815	0.904	47.3	0.691 (0.061)	1.483 (0.255)
No	E	8950	61.1	0.819	0.843	0.328	25.9	0.559 (0.048)	0.625 (0.084)
No	P	4700	54.5	0.760	2.457	1.118	30.3	0.600 (0.098)	1.713 (0.321)
No	T	6300	78.4	0.898	3.324	0.717	63.1	0.806 (0.039)	1.225 (0.179)
Yes	EPT	5450	67.3	0.837	2.815	0.921	46.9	0.689 (0.062)	1.493 (0.264)
Yes	E	7000	56.8	0.794	0.843	0.364	23.5	0.518 (0.055)	0.645 (0.086)
Yes	P	4700	55.5	0.769	2.457	1.094	33.0	0.611 (0.099)	1.646 (0.301)
Yes	T	6750	79.2	0.903	3.324	0.692	63.3	0.807 (0.039)	1.221 (0.182)

Models for each of the three orders were considerably different from each other (Table 2 and Figs 3 and 4). The only climatic variables to appear in the top five influential variables for more than one group were mean annual temperature and temperature seasonality (Figs 3 and 4).

#### Prediction of EPT richness

The EPT model explained 47.3% of the cross-validated deviance (CV correlation between raw and fitted values = 0.691 ± 0.061) in richness (Table 2). The most important variables in the EPT model were elevation and the percentage of catchment broadleaf tree cover (Table 2 and Fig 3), which both had a positive effect on richness after accounting for the average effects of all other variables (Fig 4). In general, the first five most influential variables suggest EPT richness is promoted in areas with high broadleaf tree cover, high elevation and slope, large catchment size, and low mean temperature (BIO1) (Fig 4). Latitude had little influence on the EPT model where it was included (Table 2 and S2 Fig).

#### Prediction of richness for individual orders

The Ephemeroptera model explained 25.9% of the cross-validated deviance (CV correlation = 0.559 ± 0.048) in richness (Table 2). Broadleaf tree cover was the most important influence on mayfly richness, with a similar influence on the model as in the EPT model (Table 2 and Figs 3 and 4). On the whole, the first five most influential variables on the mayfly model suggest mayfly richness is promoted in areas with high broadleaf tree cover, low PET, medium-to-high catchment sizes, high temperature seasonality (BIO4), and mean temperature of the wettest quarter (BIO8) (Fig 4). Including latitude as a predictor did not improve model fit, but the relative influence of latitude was high (19.4%), leading to a reduction in importance of climatic variables (Table 2 and S2 Fig).

The Plecoptera model was able to explain 30.3% of the cross-validated deviance (CV correlation = 0.600 ± 0.098) in richness (Table 2). Temperature seasonality (BIO4) was the most important influence on the stonefly model, with a negative influence on the response after accounting for the average effects of other variables (Table 2 and Figs 3 and 4). The first five most influential variables indicate stonefly richness is highest at sites with low temperature seasonality and mean annual temperature (BIO1), high slope and elevation, and high rainfall in the warmest quarter (BIO18) (Fig 4). Latitude contributed 10.3% to the Plecoptera model where it was included (Table 2 and S2 Fig).

The Trichoptera model explained 63.1% of the cross-validated deviance (CV correlation = 0.806 ± 0.039) in richness (Table 2). Elevation had the strongest influence on caddisfly richness after factoring out other variables (Table 2 and Figs 3 and 4). Based on the most important variables, caddisfly richness appears highest at high elevation sites, with high broadleaf tree cover, and low mean annual temperature (BIO1), temperature seasonality (BIO4) and AI. Latitude had little influence on the Trichoptera model (Table 2 and S2 Fig).

## Discussion

Environmental predictor variables of local richness differed strongly, such that no variables, either climatic, land cover or physical, were consistently important for each of the three orders, and EPT in general. Furthermore, the amount of deviance explained in richness patterns was much higher for caddisflies compared to mayflies and stoneflies. These discrepancies likely reflect differences in habitat preferences, tolerances and dispersal abilities. For instance, stoneflies are generally considered to favour colder, more oxygen rich streams [13,50], which often restricts them to headwaters, whereas the opposite may be true for mayflies and caddisflies in certain cases (e.g. [13]). While our study was influenced by several factors including clear differences between regions in stream and catchment properties, as well as different sampling dates between the regions, models had good cross-validated predictive ability. Furthermore, where season differed between sampling sites in the central, there was no difference in EPT richness.

Interestingly, while latitude had a reasonably strong influence on the Ephemeroptera and Plecoptera models, it did not improve the predictive performance of any of the models. Furthermore, latitude did not operate as expected, particularly with regard to Plecoptera. Stonefly genus richness is substantially lower in the Oriental zoogeographic zone than the Palaearctic, which the sites sampled here span the boundary of [51]. However, a reduction in richness was not evident from north to south in our study (Fig 2), and in fact the southernmost sites were the richest, reflecting more suitable habitat conditions in the southwestern sites. This latitudinal influence was less expected for mayflies and caddisflies given the minor difference in their generic richness between these two zoogeographic regions [52,53].

### Land cover influences

Given we selected sites based on minimal anthropogenic influence, we expected land cover influences to be minor on the prediction of richness. However, the most or second most important influence on EPT and mayfly (but not stonefly or caddisfly) richness was the percent of catchment broadleaf tree cover, which had a positive, and relatively linear, influence on the models. This is in line with a recent study of EPT richness in the contiguous USA [6], although their study differed from ours in that their sites covered a range of land covers including agricultural. Furthermore, across a much broader scale, Vinson & Hawkins [5] found richness of each EPT taxa was highest in broadleaf forest biomes. Forest cover in this instance likely represented a host of other factors that were associated with it, such as substrate characteristics, temperature and nutrients; a finding also suggested by Beche & Statzner [6].

As we do not expect anthropogenic pressure to be driving differences in these streams, the reason for the relatively depauperate fauna in the eastern region sites is unclear. One potential reason could be related to underlying geology. However, it may also be related to the smaller average size of these eastern sampling sites (S1 Fig). While headwaters harbor high levels of biodiversity, this is often at the beta diversity level through turnover between sites rather than at the alpha diversity level [54–56]. This may be a result of lower connectivity in headwaters than downstream sections, leading to a possible transition from species sorting in headwaters to mass effects downstream [57]. This should result in higher alpha diversity in downstream sections compared to headwaters and vice versa for beta diversity.

### Physical influences

We expected elevation to be an important predictor of richness given the large range between the three regions (see S1 Fig). However, while elevation strongly influenced the BRT models for caddisflies and total EPT, it had a minor influence on mayflies and stoneflies. This was mostly evident as an increase from 0–1000 m asl. Previous studies on stream invertebrates have found decreasing richness with elevation [58,59], while in our study, elevation had a positive influence on the EPT and caddisfly richness models. While stonefly richness clearly increased from east to west, elevation did not have a strong influence on their prediction. Nonetheless, when assessing the link with elevation separately, richness also increased significantly for stoneflies (S3 Fig), which somewhat fits with their previously stated temperature preferences. There was also evidence of a mid-domain effect (i.e. peak at intermediate elevation) for mayflies (S3 Fig), although this may be an artifact of the data related to reduced richness in the eastern region streams, which were also lower in elevation than the other sites.

The lack of influence of elevation likely reflects the fact that temperature and slope, which covary strongly with elevation, are more important factors than elevation itself. Therefore, elevational changes often affect species through indirect changes to factors such as temperature. The benefits of the approach we used here (BRTs) is clearly emphasized with its ability to tease apart the key and potentially more direct influences on richness as opposed to the more indirect influence of elevation. For instance, slope was a good predictor of stonefly richness, which fits with expectations for the preferences for cool, high-oxygenated streams. Nevertheless, it is important to bear in mind that streams were clustered in three distinct regions, which differed in their characteristics, such as elevation and slope, as well as sampling approach, giving rise to spatial autocorrelation in some predictor variables.

### Climatic influences

Of the climatic drivers, temperature mean and seasonality were the most consistently important for all groups, with organisms favouring less arid areas with lower mean temperatures and seasonality. This trend was usually apparent in the middle of the range of temperature related variables. We expected temperature to play a major role in predicting richness due to species richness being more closely correlated with energy availability in regions without water deficit [60,61]. However, important temperature variables suggested cooler and less extreme (i.e. lower PET, AI, BIO1, BIO4) areas supported higher richness and their influence was variable across the three groups.

Given productivity and disturbance can have strong and often interactive effects on biodiversity patterns, both in general [62,63] and in streams [64–66], we expected that variables associated with disturbance (e.g. precipitation seasonality), despite being seasonally predictable in monsoonal areas, and productivity (e.g. PET and temperature of wettest quarter) may have important influences on richness patterns. This proxy-based approach was used by Vinson & Hawkins [5], who found productivity and disturbance strongly explained global patterns in stream insect richness. However, climatic factors that were consistently more important in our study represented temperature, rather than rainfall, seasonality and extremes (e.g. temperature seasonality and PET), which likely reflects the smaller spatial scale of our study compared to Vinson & Hawkins [5]. Models at the catchment scale (< 2000 km^2^) in Chinese streams have found that seasonal hydrological events triggered by monsoonal precipitation are important predictors of species distributions of stream macroinvertebrates [31].

### Historical and scale-related influences

While contemporary ecological factors may be more influential on stream insect diversity patterns than historical biogeographical influences (e.g. [5]), it is important to consider that historical land use may influence the present day biodiversity pattern [8]. Despite selecting for low impact sites based on the present-day conditions, the long history of Chinese civilization suggests there may be a historical land use signature present in these streams through centuries of use (mainly rice and tea plantations in the central and eastern zones and grazing in the west) that we were unable to determine at the site selection phase.

The factor ‘region’, which can be thought of as somewhat representing historical conditions, had little influence on the outcome of the BRT models, although many environmental variables differed strongly between the three regions. For instance, streams in the east were smaller (based on catchment size) and many other physicochemical variables differed (S1 Fig). A previous study on macroinvertebrate communities across a large spatial scale in China found spatial variation between locations was the best predictor of communities, with communities grouping based on location rather than some other environmental gradient [28]. The influence of local and regional influences on richness in streams varies with scale [19], and clear evidence of this scale-dependence exists in China for woody plants, for example [67]. The inclusion of local-scale variables would likely have increased the predictive ability of models, as these are essentially finer resolution reflections of larger-scale influences. For instance, a recent study in pristine New Zealand streams found high levels of variation in EPT richness could be explained from variables limited to the local scale (i.e. variables limited to the sampling reach, rather than catchment based) [68].

Traditionally, but somewhat anecdotally, stoneflies have been considered weaker dispersers than other groups of stream insects (e.g. [69]), thus one might expect historical influences to shape their distribution more significantly than the other groups. However, as China has experienced a relatively benign climatic history, not experiencing glaciation during the Last Glacial Maximum (LGM), with the exception of the Qinghai-Tibet-Plateau, we did not expect strong dispersal limitation to be a major factor shaping these stream assemblages. In fact, niche/environmental factors were found to exert more control on EPT richness in contiguous USA streams than spatial factors suggesting little dispersal limitation for these taxa [6]. Furthermore, Heino and Mykrä [70] found no difference in the spatial structuring of mayflies, stoneflies, caddisflies and midges, which are considered to differ substantially in dispersal ability. Nonetheless, historical influences may have had an influence on patterns with regard to the higher stonefly richness in western sites. Others have found high richness in this Eastern Himalayas region [71], known as a biodiversity hotspot. Higher stonefly richness in this region may be related to their weaker dispersal ability leading to greater species accumulation than the other areas as a result of the dynamic historical rising processes of the Qinghai-Tibetan Plateau [72].

## Conclusions

The relative importance of climate, land cover, and physical factors on stream insect richness varied considerably between the three orders across these mid-latitude Chinese streams. No specific variables were consistently strong predictors of richness of the three orders or combined EPT, with broadleaf tree cover, elevation, slope, temperature seasonality and mean temperature being the most important. Furthermore, while latitude was an important predictor of Ephemeroptera and Plecoptera richness, it did not improve overall prediction of any of the groups. Given the lack of large-scale assessments of stream insect richness across China, this research opens the door for more in-depth research across broader spatial scales. However, for this to be possible it is crucial that data be made available and collection methods are consistent across studies.

## Supporting Information

S1 Fig Summary of environmental variables. Differences between the three regions for each environmental variable for 80 streams across mid-latitude China. Autocorrelated bioclimatic variables (*r* > 0.75) have been removed. Results of ANOVA testing for differences between regions are indicated in the title of each plot as follows: * = *P* < 0.05, ** = *P* < 0.01, *** = *P* < 0.0001, blank = no significant difference. BIO values are BIOCLIM parameters. Temperature values are measured in °C and multiplied by 10, precipitation values are in mm. BIO1 = Annual Mean Temperature; BIO4 = Temperature Seasonality (SD x 100); BIO8 = Mean Temperature of Wettest Quarter; BIO15 = Precipitation Seasonality (Coefficient of Variation); BIO18 = Precipitation of Warmest Quarter; PET = Potential Evapo-transpiration; AI = Aridity Index; Trees (BL) = % catchment broadleaf trees; Trees (NL) = % catchment needleleaf trees; Shrub = % catchment shrub vegetation; Herbaceous = % catchment herbaceous vegetation; Cultivated = % catchment cultivated land cover; Water = % catchment water; Elevation = Elevation in m asl; Catchment size = Catchment size in km^2^; Slope = Catchment slope in degrees.
(EPS)

S2 Fig Relative influence of variables including latitude on richness patterns. Relative influence of each of the variables (climate, land cover and physical) with latitude included as a predictor variable on each of the four boosted regression tree models predicting Ephemeroptera (E), Plecoptera (P), Trichoptera (T) and combined EPT richness taken from 80 streams across mid-latitude China. Explanations and units of variables are given in Table 1 in the main text.
(EPS)

S3 Fig Elevation-richness relationships. Ephemeroptera (E), Plecoptera (P), Trichoptera (T) and combined EPT richness (mostly genus level) as a function of log-transformed elevation in m a.s.l. in 80 streams across mid-latitude China. Regression results are given with each graph.
(EPS)

## References

[1] Currie DJ (1991) Energy and large-scale patterns of animal-species and plant-species richness. American Naturalist 137: 27–49.

[2] Gaston KJ (2000) Global patterns in biodiversity. Nature 405: 220–227. 10821282 10.1038/35012228

[3] Ricklefs RE (2004) A comprehensive framework for global patterns in biodiversity. Ecology Letters 7: 1–15.

[4] Willig MR, Kaufman DM, Stevens RD (2003) Latitudinal gradients of biodiversity: pattern, process, scale, and synthesis. Annual Review of Ecology, Evolution, and Systematics 34: 273–309.

[5] Vinson MR, Hawkins CP (2003) Broad-scale geographical patterns in local stream insect genera richness. Ecography 26: 751–767.

[6] Bêche LA, Statzner B (2009) Richness gradients of stream invertebrates across the USA: taxonomy- and trait-based approaches. Biodiversity and Conservation 18: 3909–3930.

[7] Heino J (2009) Biodiversity of aquatic insects: spatial gradients and environmental correlates of assemblage-level measures at large scales. Freshwater Reviews 2: 1–29.

[8] Harding JS, Benfield EF, Bolstad PV, Jones EBD III (1998) Stream biodiversity: The ghost of land use past. Proceedings of the National Academy of Sciences 95: 14843–14847. 9843977 10.1073/pnas.95.25.14843 PMC24537

[9] Allan J (2004) Landscapes and riverscapes: the influence of land use on stream ecosystems. Annual Review of Ecology, Evolution, and Systematics 35: 257–284.

[10] Tonkin JD, Death RG, Barquín J (2013) Productivity–diversity relationships for stream invertebrates differ geographically. Aquatic Ecology 47: 109–121.

[11] Heino J, Virkkala R, Toivonen H (2009) Climate change and freshwater biodiversity: detected patterns, future trends and adaptations in northern regions. Biological Reviews 84: 39–54. 10.1111/j.1469-185X.2008.00060.x 19032595

[12] Poff NL, Allan JD, Bain MB, Karr JR, Prestegaard KL, Richter BD, et al (1997) The Natural Flow Regime. Bioscience 47: 769–784.

[13] Haidekker A, Hering D (2007) Relationship between benthic insects (Ephemeroptera, Plecoptera, Coleoptera, Trichoptera) and temperature in small and medium-sized streams in Germany: A multivariate study. Aquatic Ecology 42: 463–481.

[14] Vinson MR, Hawkins CP (1998) Biodiversity of stream insects: variation at local, basin, and regional scales. Annual Review of Entomology 43: 271–293. 15012391 10.1146/annurev.ento.43.1.271

[15] Tonkin JD, Stoll S, Sundermann A, Haase P (2014) Dispersal distance and the pool of taxa, but not barriers, determine the colonisation of restored river reaches by benthic invertebrates. Freshwater Biology 59: 1843–1855.

[16] Brosse S, Arbuckle CJ, Townsend CR (2003) Habitat scale and biodiversity: influence of catchment, stream reach and bedform scales on local invertebrate diversity. Biodiversity and Conservation 12: 2057–2075.

[17] Heino J, Muotka T, Paavola R (2003) Determinants of macroinvertebrate diversity in headwater streams: regional and local influences. Journal of Animal Ecology 72: 425–434.

[18] Astorga A, Heino J, Luoto M, Muotka T (2011) Freshwater biodiversity at regional extent: determinants of macroinvertebrate taxonomic richness in headwater streams. Ecography 34: 705–713.

[19] Mykrä H, Heino J, Muotka T (2007) Scale-related patterns in the spatial and environmental components of stream macroinvertebrate assemblage variation. Global Ecology and Biogeography 16: 149–159.

[20] Poff NL (1997) Landscape filters and species traits: Towards mechanistic understanding and prediction in stream ecology. Journal of the North American Benthological Society 16: 391–409.

[21] Fu C, Wu J, Chen J, Wu Q, Lei G (2003) Freshwater fish biodiversity in the Yangtze River basin of China: patterns, threats and conservation: 1649–1685.

[22] Tang Z, Wang Z, Zheng C, Fang J (2006) Biodiversity in China’s mountains. Frontiers in Ecology and the Environment 4: 347–352.

[23] Fang J, Shen Z, Tang Z, Wang X, Wang Z, Feng J, et al (2012) Forest community survey and the structural characteristics of forests in China. Ecography 35: 1059–1071.

[24] Liu J, Tian H, Liu M, Zhuang D, Melillo JM (2005) China’s changing landscape during the 1990s: Large-scale land transformations estimated with satellite data. Geophysical Research Lettersy 32: 1–5.

[25] Zhao S, Peng C, Jiang H, Tian D, Lei X, Zhou X (2006) Land use change in Asia and the ecological consequences. Ecological Research 21: 890–896.

[26] Li F, Cai Q, Ye L (2010) Developing a Benthic Index of Biological Integrity and some relationships to environmental factors in the subtropical Xiangxi River, China. International Review of Hydrobiology 95: 171–189.

[27] Wang X, Cai Q, Tang T, Yang S, Li F (2011) Spatial distribution of benthic macroinvertebrates in the Erhai basin of southwestern China. Journal of Freshwater Ecology 27: 37–41.

[28] Li F, Cai Q, Qu X, Tang T, Wu N, Fu X, et al (2012) Characterizing macroinvertebrate communities across China: Large-scale implementation of a self-organizing map. Ecological Indicators 23: 394–401.

[29] Lenat DR (1988) Water quality assessment of streams using a qualitative collection method for benthic invertebrates. Journal of the North American Benthological Society 7: 222–233.

[30] Schmalz B, Kuemmerlen M, Kiesel J, Jähnig SC, Fohrer N (2014) Impacts of land use change on hydrological components and macroinvertebrate distributions in the Poyang lake area. Ecohydrology: 10.1002/eco.1569

[31] Kuemmerlen M, Domisch S, Schmalz B, Cai Q (2012) Integrierte Modellierung von aquatischen Ökosystemen in China: Arealbestimmung von Makrozoobenthos auf Einzugsgebietsebene. Hydrologie und Wasserbewirtschaftung 56: 185–192.

[32] Jähnig SC, Cai Q (2010) River water quality assessment in selected Yangtze tributaries: Background and method development. Journal of Earth Science 21: 876–881.

[33] Barbour MT, Stribling JB, Verdonschot PFM (2006) The multihabitat approach of USEPA’s rapid bioassessment protocols: benthic macroinvertebrates. Limnetica 25: 839–850.

[34] Morse JC, Yang L, Tian L (1994) Aquatic Insects of China Useful for Monitoring Water Quality. 1st ed. Morse JC, Yang L, Tian L, editors Nanjing: Hohai University Press.

[35] Dudgeon D (1999) Tropical Asian Streams: Zoobenthos, Ecology and Conservation. 1st ed. Hong Kong: Hong Kong University Press.

[36] Yule CM, Yong H (2004) Freshwater Invertebrates of the Malaysian Region. Yule CM, Yong H, editors Malaysia: Academy of Sciences Malaysia.

[37] Mekong River Commission (2006) Identification of Freshwater Invertebrates of the Mekong River and its Tributaries. Vientiane, Lao PDR: Mekong River Commission.

[38] Nesemann H, Sharma S, Sharma G, Khanal S, Pradhan B, Shah DN, et al (2007) Aquatic Invertebrates of the Ganga River System. H. Nesemann.

[39] Lehner B, Verdin K, Jarvis A (2006) HydroSHEDS Technical Documentation. Washington DC.

[40] Hijmans RJ, Cameron SE, Parra JL, Jones PG, Jarvis A (2005) Very high resolution interpolated climate surfaces for global land areas. International Journal of Climatology 25: 1965–1978.

[41] Zomer RJ, Trabucco A, Bossio DA, Verchot LV (2008) Climate change mitigation: A spatial analysis of global land suitability for clean development mechanism afforestation and reforestation. Agriculture, Ecosystems & Environment 126: 67–80.

[42] Kuemmerlen M, Schmalz B, Guse B, Cai Q, Fohrer N, Jähnig SC (2014) Integrating catchment properties in small scale species distribution models of stream macroinvertebrates. Ecological Modelling 277: 77–86.

[43] R Core Team (2013) R: A language and environment for statistical computing R Foundation of Statistical Computing, Vienna, Austria.

[44] Hothorn T, Bretz F, Westfall P (2008) Simultaneous inference in general parametric models. Biometrical Journal 50: 346–363. 10.1002/bimj.200810425 18481363

[45] Hastie T, Tibshirani R, Friedman J (2009) Elements of Statistical Learning: Data Mining, Inference and Prediction. Springer, New York 10.1016/j.neunet.2009.04.005

[46] Friedman J, Hastie T, Tibshirani R (2000) Additive logistic regression: a statistical view of boosting. The Annals of Statistics 28: 337–374.

[47] Elith J, Leathwick JR, Hastie T (2008) A working guide to boosted regression trees. Journal of Animal Ecology 77: 802–813. 10.1111/j.1365-2656.2008.01390.x 18397250

[48] Hijmans RJ, Phillips S, Leathwick J, Elith J (2013) dismo: Species distribution modeling. R package version 0.9–3. http://CRAN.R-project.org/package=dismo.

[49] Buston PM, Elith J (2011) Determinants of reproductive success in dominant pairs of clownfish: a boosted regression tree analysis. Journal of Animal Ecology 80: 528–538. 10.1111/j.1365-2656.2011.01803.x 21284624

[50] Tierno de Figueroa JM, López-Rodríguez MJ, Lorenz A, Graf W, Schmidt-Kloiber A, Hering D (2009) Vulnerable taxa of European Plecoptera (Insecta) in the context of climate change. Biodiversity and Conservation 19: 1269–1277.

[51] Fochetti R, Tierno de Figueroa J (2008) Global diversity of stoneflies (Plecoptera; Insecta) in freshwater. Hydrobiologia 595: 365–377.

[52] De Moor FC, Ivanov VD (2007) Global diversity of caddisflies (Trichoptera: Insecta) in freshwater. Hydrobiologia 595: 393–407.

[53] Barber-James HM, Gattolliat J-L, Sartori M, Hubbard MD (2007) Global diversity of mayflies (Ephemeroptera, Insecta) in freshwater. Hydrobiologia 595: 339–350.

[54] Finn DS, Bonada N, Múrria C, Hughes JM (2011) Small but mighty: headwaters are vital to stream network biodiversity at two levels of organization. Journal of the North American Benthological Society 30: 963–980.

[55] Besemer K, Singer G, Quince C, Bertuzzo E, Sloan W, Battin TJ (2013) Headwaters are critical reservoirs of microbial diversity for fluvial networks. Proceedings of The Royal Society B 280: 20131760 10.1098/rspb.2013.1760 24089333 PMC3790480

[56] Clarke A, Mac Nally R, Bond N, Lake PS (2008) Macroinvertebrate diversity in headwater streams: a review. Freshwater Biology 53: 1707–1721.

[57] Brown BL, Swan CM (2010) Dendritic network structure constrains metacommunity properties in riverine ecosystems. Journal of Animal Ecology 79: 571–580. 10.1111/j.1365-2656.2010.01668.x 20180874

[58] Jacobsen D (2004) Contrasting patterns in local and zonal family richness of stream invertebrates along an Andean altitudinal gradient. Freshwater Biology 49: 1293–1305.

[59] Jacobsen D (2008) Low oxygen pressure as a driving factor for the altitudinal decline in taxon richness of stream macroinvertebrates. Oecologia 154: 795–807. 17960424 10.1007/s00442-007-0877-x

[60] Pausas JG, Austin MP (2001) Patterns of plant species richness in relation to different environments: An appraisal. Journal of Vegetation Science 12: 153–166.

[61] Kreft H, Jetz W (2007) Global patterns and determinants of vascular plant diversity. Proceedings of the National Academy of Sciences of the United States of America 104: 5925–5930. 17379667 10.1073/pnas.0608361104 PMC1851593

[62] Huston M (1994) Biological Diversity: The Coexistence of Species on Changing Landscapes. Cambridge, UK: Cambridge University Press.

[63] Kondoh M (2001) Unifying the relationships of species richness to productivity and disturbance. Proceedings of the Royal Society of London Series B-Biological Sciences 268: 269–271. 11217897 10.1098/rspb.2000.1384 PMC1088602

[64] Tonkin JD, Death RG (2012) Consistent effects of productivity and disturbance on diversity between landscapes. Ecosphere 3: art108. 24371541

[65] Tonkin JD, Death RG (2013) Scale dependent effects of productivity and disturbance on diversity in streams. Fundamental and Applied Limnology / Archiv für Hydrobiologie 182: 283–295.

[66] Tonkin JD, Death RG, Collier KJ (2013) Do productivity and disturbance interact to modulate macroinvertebrate diversity in streams? Hydrobiologia 701: 159–172. 10.1016/j.ejphar.2012.12.031 23340222

[67] Wang Z, Rahbek C, Fang J (2012) Effects of geographical extent on the determinants of woody plant diversity. Ecography 35: 1160–1167.

[68] Tonkin JD (2014) Drivers of macroinvertebrate community structure in unmodified streams. PeerJ 2: e465 10.7717/peerj.465 25024926 PMC4081181

[69] Brittain JE (1990) Life history strategies in Ephemeroptera and Plecoptera In: Campbell J, editor. Mayflies and stoneflies. Kluwer pp. 1–12.

[70] Heino J, Mykrä H (2008) Control of stream insect assemblages: roles of spatial configuration and local environmental factors. Ecological Entomology 33: 614–622.

[71] Wang Z, Fang J, Tang Z, Lin X (2012) Relative role of contemporary environment versus history in shaping diversity patterns of China’s woody plants. Ecography 35: 1124–1133.

[72] Favre A, Päckert M, Pauls SU, Jähnig SC, Uhl D, Michalak I (2014) The role of the uplift of the Qinghai-Tibetan Plateau for the evolution of Tibetan biotas. Biological Reviews 10.1111/brv.12107 24784793

